# Usefulness of limited sampling strategy for mycophenolic acid area under the curve considering postoperative days in living-donor renal transplant recipients with concomitant prolonged-release tacrolimus

**DOI:** 10.1186/s40780-017-0086-7

**Published:** 2017-06-24

**Authors:** Tomoyuki Enokiya, Kouhei Nishikawa, Yuichi Muraki, Takuya Iwamoto, Hideki Kanda, Yoshiki Sugimura, Masahiro Okuda

**Affiliations:** 1 Department of Pharmacy, Mie University Hospital, Faculty of Medicine, Mie University, 2-174 Edobashi, Tsu, Mie 514-8507 Japan; 2 Department of Nephro-Urologic Surgery and Andrology, Mie University Hospital, Mie University, Tsu, Mie 514-8507 Japan

**Keywords:** Mycophenolate mofetil, Limited sampling strategy, Living-donor renal transplantation, Mycophenolic acid, Therapeutic drug monitoring

## Abstract

**Background:**

The optimal dose of mycophenolate mofetil (MMF) in renal transplant patients has been recommended to be decided on the basis of area under the concentration-time curve (AUC_0-12_) of mycophenolic acid (MPA). Although meta-analysis has revealed that postoperative day (POD) is an influencing factor in MPA pharmacokinetics, there are no reports regarding a limited sampling strategy (LSS) for MPA AUC in consideration of POD. The aim of this study was to construct of an LSS considering POD that appropriately expresses the MPA AUC following renal transplantation and evaluation of the usefulness.

**Methods:**

Serum concentration–time profiles (measured AUC_0-12_) comprising nine sampling points over 12 h were analyzed in 36 living-donor renal transplant recipients after MMF administration with concomitant once-daily prolonged-release tacrolimus. Two LSSs were developed by stepwise multiple regression analysis (Method A: not classified by PODs; Method B: classified by PODs into POD < 31 and POD ≥ 31). Each LSS comprised four blood-sampling points within 6 h after MMF administration. Precision and reliability were verified by using root-mean-square error (RMSE), correlation coefficient (R^2^), and coefficient of determination (q^2^) by using leave-one-out cross-validation. The absolute values of the difference between measured and estimated AUCs (delta AUC) were compared for both estimating equations.

**Results:**

One-hundred samples obtained from 36 recipients for AUC_0-12_ comprised POD < 31 (*n* = 39) and POD ≥ 31 (*n* = 61). Estimation of AUC_0-12_ by Method B resulted in better accuracy and reliability (Method A: RMSE = 5.5, R^2^ = 0.85, q^2^ = 0.83; Method B: POD < 31: RMSE = 5.5, R^2^ = 0.86, q^2^ = 0.83; POD ≥ 31: RMSE = 3.9, R^2^ = 0.92, q^2^ = 0.89) and significantly lower median delta AUC compared with that by Method A (delta AUC: 2.6 (0.0–11.6) v.s. 3.9 (0.1–18.1), *p* = 0.032).

**Conclusion:**

These results suggest that LSS, classified as POD < 31 or POD > 31, would provide more accurate and reliable estimation of MPA AUC_0-12_ in Japanese living-donor renal transplant patients.

**Electronic supplementary material:**

The online version of this article (doi:10.1186/s40780-017-0086-7) contains supplementary material, which is available to authorized users.

## Background

Mycophenolate mofetil (MMF), a prodrug of the immunosuppressant mycophenolic acid (MPA), has been widely used for the prevention of rejection in solid organ transplant patients [1–3]. MMF is administered to patients who have undergone renal transplantation at a dosage of 0.5–1.5 g twice daily. After oral administration, MMF is rapidly absorbed and hydrolyzed to MPA [4], and is then inactivated to MPA glucuronide by UDP-glucuronosyltransferase [5].

Numerous studies have demonstrated the relationship between area under the concentration-time curve (AUC_0-12_) of MPA and both risk of rejection [4, 6–12] and hematologic side effects [11, 13]. A target range of 30–60 mg h/L for the MPA AUC_0-12_ has been proposed as a guide to MMF dosage in renal transplant patients in these studies. There were large inter- and intra-individual variations in MPA AUC_0-12_ [13]. However, routine measurement of full MPA AUC_0-12_ for 12-h dose intervals is cumbersome and cost-prohibitive. Limited sampling strategies (LSSs) have been developed in several countries for estimating MPA AUC_0-12_ to overcome these difficulties [14].

van Hest et al. [15] reported that MPA pharmacokinetics are affected by the patient’s renal function, serum albumin concentration, and dosage of immunosuppressants, including calcineurin inhibitors that are dependent on the post-renal transplant period. Moreover, several studies reported that oral MPA clearance is inversely proportional to postoperative days (POD), achieving gradual stability [7, 11]. Therefore, sampling points to estimate MPA AUC_0-12_ might vary according to POD. However, there is no report demonstrating LSS design with consideration of POD. The aim of this study was to develop LSS with consideration of POD, and to evaluation of the usefulness of these LSSs in Japanese renal transplant patients.

## Methods

### Patients

This study was performed on all 36 patients who underwent living-donor renal transplantation at Mie University Hospital between November 2005 and August 2015. One-hundred serum MPA concentration–time profiles were prospectively obtained between November 2012 and September 2015.

### Data collection

Demographic data including concomitant drug use were obtained by reviewing electronic medical records of the patients. Concomitant drugs that are documented in Lexicomp, integrated with UpToDate (version 2014; Wolters Kluwer Health, Philadelphia, PA, USA) were considered.

### Assay of serum MPA concentration

Serum was separated by centrifugation at 1700 × *g* for 10 min by using serum separation tubes. Serum MPA concentration was determined by using a homogeneous particle-enhanced turbidimetric inhibition immunoassay (PETINIA) technique on a DIMENSION® Xpand Plus Integrated Chemistry System (Siemens Healthcare Diagnostics K.K., Tokyo, Japan).

### Immunosuppression regimen

All patients received a basic immunosuppression regimen of MMF (CellCept; Chugai Pharmaceutical Co., Ltd., Tokyo, Japan), once-daily prolonged-release tacrolimus (Graceptor; Astellas, Tokyo, Japan,), methylprednisolone, and basiliximab (Simulect i.v. injection; Novartis Pharmaceuticals, Tokyo, Japan). In addition, patients with blood type incompatibility received rituximab at a dose of 200 mg on preoperative day 4. The MMF was taken on preoperative day 4 at a fixed starting dose of 0.5 g twice daily, 1 g twice daily from POD 0, 0.75 g twice daily from POD 15, and 0.5 g twice daily from POD 60. The starting dose of tacrolimus was 0.1 mg/kg/day, adjusted based on whole-blood concentration (target concentration: 6.0–8.0 ng/mL). Methylprednisolone was started at a dose of 20 mg/day and administered at a dose of 250 mg during surgery, reduced gradually to the maintenance dose of 4 mg/day from POD 30. Basiliximab was injected intravenously at a dose of 20 mg within 2 h before the operation and on POD 4.

### Determination of MPA AUC_0-12_

Serum MPA concentration was determined just before dosage and at 0.5, 1, 2, 3, 4, 6, 8, and 12 h after administration. The MPA AUC_0-12_ was calculated using a linear trapezoidal rule. The AUC_0-12_ of MPA was determined on POD 7, POD 14, POD 21–28, and POD ≥ 31 according to recommendation on the application of therapeutic drug monitoring to MMF therapy in transplantation [16].

### Development of a POD-based LSS

One-hundred serum MPA concentration–time profiles were classified into four groups on the basis of POD (POD 7, POD 14, POD 21–28 and POD ≥ 31). MPA clearance (oral MMF dose per AUC_0-12_) was compared between these four groups by using multiple comparison test. POD-based LSSs were developed using multiple comparison analysis. Each estimating equation was developed by using stepwise multiple regression analysis, and comprised four blood-sampling points over 6 h after drug administration.

### Evaluation of estimating equations

Precision was evaluated by using Spearman’s rank correlation test, root-mean-square error (RMSE), correlation coefficient (R^2^) by least squares method, and correlation between measured AUC and estimated AUC. Reliability was evaluated by using coefficient of determination (q^2^), calculated using leave-one-out cross-validation (LOOCV).

The absolute value of the difference (delta AUC) between measured and estimated AUCs was compared between each LSS by using the Wilcoxon matched pair test.

### Statistical analysis

Spearman’s rank correlation test, RMSE, and least squares method were performed using JMP® Ver. 7.0 (SAS Institute, Cary, NC, USA). Wilcoxon matched pair and Kruskal-Wallis tests were performed using GraphPad Prism Ver. 5.01 (GraphPad Software, Inc., San Diego, CA, USA). LOOCV and multiple comparisons by Wilcoxon signed-rank test (Holm’s method) were performed using GNU R Ver. 3.1.0 for windows. A *P* value < 0.05 was considered significant.

## Results

### Development of POD-based LSS

**Fig. 1:**
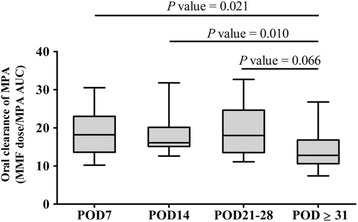
Differences in clearance of mycophenolic acid in postoperative periods. Graphs display median and interquartile range. AUC: area under the concentration-time curve; POD: postoperative day. Number of actual AUC_0-12_ of mycophenolic acid: 13 (POD 7), 16 (POD 14), 10 (POD 21–28), 61 (POD ≥ 31)

The AUCs_0-12_ of MPA on POD 7 (*n* = 13), POD 14 (*n* = 16), POD 21–28 (*n* = 10), and POD ≥ 31 (*n* = 61) were determined as described in the materials and methods section. Significant differences in the oral clearance of MPA were found between POD ≥ 31 and POD 7 or POD 14 by multiple comparison analysis (Fig. 1). Moreover, MPA oral clearance tended to be lower in POD ≥ 31 compared with that in POD 21–28. There were no significant differences in MPA oral clearance between POD 7, POD 14, and POD 21–28. On the basis of these results, the LSSs for PODs < 31 or ≥ 31 were defined by using Method B.

### Demographic data

**Fig. 2:**
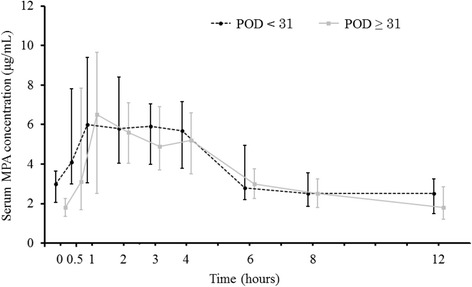
Median (interquartile range) serum concentration–time profiles of MPA. *Block circle* points on *broken line* depict POD < 31 and *gray* *square* points on *solid line* depict POD ≥ 31 MPA: mycophenolic acid; POD: postoperative day

Table 1 displays the data on patient characteristics including gender, primary disease, age, body weight, creatinine clearance estimated by Cockcroft and Gault formula, alanine aminotransferase, aspartate aminotransferase, total bilirubin, concomitant drugs, and measured MPA AUC_0-12_. Figure 2 shows the median concentration–time profile. Median (range) C0, t_max_, and C_max_ were 2.9 (0.3–7.7) μg/mL, 2.3 (0.5–6.0) h, and 11.3 (3.3–27.3) μg/mL for POD < 31, and 1.9 (0.2–5.7) μg/mL, 2.2 (0.5–6.0) h, and 11.9 (3.4–43.2) μg/mL for POD ≥ 31, respectively. Median concentrations of MPA for POD ≥ 31 during 1 and 4 h after administration were mostly constant similarly as those for POD < 31 (Fig. 2). In contrast, the AUC_0-12_ of MPA per dose (mg) for POD < 31 was significantly lower than that for POD ≥ 31 (median (range): 0.056 (0.031–0.098) v.s. 0.078 (0.029–0.155), *P* < 0.0001).

**Table 1:** Demographic data in living donor renal transplant recipients

	The number of patients (%) or median [minimum–maximum]
Male	17 (47.2)
Primary disease	
diabetic nephropathy	9 (25.0)
IgA nephropathy	7 (19.4)
polycystic kidney	3 (8.3)
chronic glomerulonephritis	2 (5.5)
focal glomerulosclerosis	2 (5.5)
Alport syndrome	1 (2.7)
cystinosis	1 (2.7)
mesangial proliferative glomerulonephritis	1 (2.7)
unknown	10 (26.0)
Age^a^	47 [28–66]
Body weight (kg)^a^	55.5 [34.8–105.9]
Serum albumin (g/dL)^a^	4.0 [2.9–5.1]
Serum creatinine (mg/dL)^a^	1.1 [0.5–2.9]
Estimated creatinine clearance (mL/min)^a^	57.5 [24.9–113.8]
Total bilirubin (mg/dL)^a^	0.6 [0.2–1.6]
Alanine aminotransferase (IU/mL)^a^	12 [4–117]
Aspartate aminotransferase (IU/mL)^a^	17 [6–109]
Mycophenolate mofetil dose at one time (mg)	
Postoperative day < 31	1000 [500–1000]
Postoperative day ≥ 31	500 [250–1000]
Actual AUC_0-12_ of mycophenolic acid (μg▪h/mL)	
Postoperative day < 31	52.7 [23.6–89.2]
Postoperative day ≥ 31	43.7 [21.5–87.6]
Oral clearance of mycophenolate acid	
Postoperative day < 31	18.0 [10.2–32.7]
Postoperative day ≥ 31	12.5 [6.4–34.1]
Postoperative day	
Postoperative day < 31	14 [7–21]
Postoperative day ≥ 31	359 [34–2832]
Concomitant drug^a ,b^	
Postoperative day < 31	
Proton pump inhibitor	34 (34.0)
Proton pump inhibitor + Quinolone	4 (4.0)
Postoperative day ≥ 31	
Quinolone	22 (22.0)
Proton pump inhibitor	16 (16.0)
Proton pump inhibitor + Quinolone	17 (17.0)
Proton pump inhibitor + Valganciclovir	2 (2.0)

^a^These data were measured on the day the AUC_0-12_ was determined (*n* = 100)

^b^Concomitant drugs that may influence MPA pharmacokinetics were examined by using Lexicomp© integrated in UpToDate^©^(version 2014; Wolters Kluwer Health, Philadelphia, PA, USA)

### Evaluation of estimating equations

**Fig. 3:**
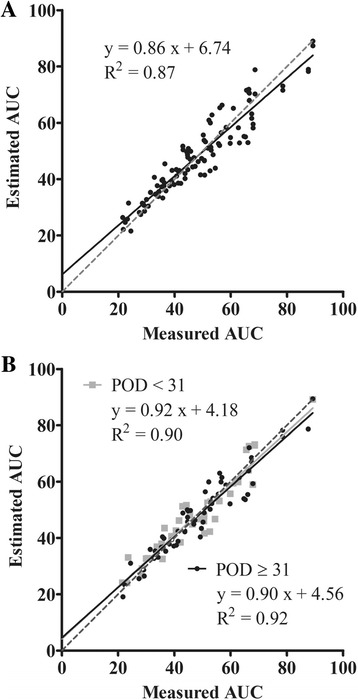
Correlation between measured and estimated AUC_0-12_ of mycophenolic acid for two limited sampling strategies. **a**: Method A (*n* = 100); **b**: Method B (*gray square* and *solid line*: POD < 31 (*n* = 39), *black circle* and *solid line*: POD ≥ 31 (*n* = 61)). AUC: area under the concentration-time curve. *Dotted lines* shows 1:1 correlation

**Fig. 4:**
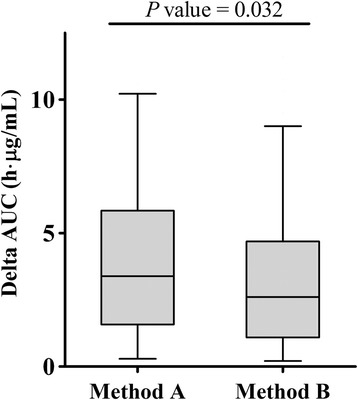
Comparison of delta AUC_0-12_ (absolute value of discrepancy between measured and estimated AUC) for two limited sampling strategies. Graph shows median and interquartile range. AUC: area under the concentration-time curve

The *P* value, RMSE, R^2^, and q^2^ of each estimating equation are shown in Table 2. The *P* values of all estimating equations were less than 0.001. The worst precision (RMSE and R^2^) and reliability (q^2^) were observed in MPA AUC_0-12_ estimation of POD < 31 by Method A. Estimation by Method B resulted in better precision (RMSE and R^2^) and reliability (q^2^) than that by Method A. The correlation of Method B with estimated and measured AUC was better than that of Method A, as demonstrated by the results where corresponding slope, intercept, and R^2^ values of Method B were much closer to 1, 0, and 1, respectively (Fig. 3). Wilcoxon matched pair test indicated that delta AUC estimated by Method B was significantly lower than that by Method A (Fig. 4).

**Table 2:** Correlation with measured AUC_0-12_, accuracy, and reliability of each estimated formula

		n	Equations for AUC_0-12_ estimation	*P* value^*^	RMSE^**^	R^2#^	q^2 ##^
Method A	All patients	100	7.4 + 2.3 × C_0h_ + 1.2 × C_1h_ + 2.3 × C_3h_ + 4.4 × C_6h_	<0.0001	5.5	0.85	0.83
Method B	POD < 31	39	10.6 + 1.1 × C_1h_ + 1.1 × C_2h_ + 2.0 × C_4h_ + 3.9 × C_6h_	< 0.0001	5.5	0.86	0.83
	POD ≥ 31	61	3.8 + 3.5 × C_0h_ + 1.2 × C_1h_ + 1.9 × C_3h_ + 5.4 × C_6h_	< 0.0001	3.9	0.92	0.89

*C*_*time*_ serum mycophenolic acid concentration at time after administration, *POD* postoperative day

*P value: Spearman’s rank correlation test, **RMSE root-mean-square error, ^#^ *R* ^*2*^ least squares method, ^##^ *q* ^*2*^ leave-one-out cross-validation

## Discussion

In this study, two different approaches to developing LSSs for the estimation of AUC_0-12_ were evaluated by statistical analyses (Method A: not classified by POD, Method B: classified by POD into < 31 or ≥ 31 based on difference in MPA systemic clearance). Precision (RMSE and R^2^) and reliability (q^2^) were compared between Method A and Method B, and Method B provided better estimation of AUC_0-12_ compared with Method A. Moreover, delta AUC of Method B was lower than that of Method A. Therefore, these results suggested that LSSs considering POD would provide more precise and reliable estimation of MPA AUC_0-12_.

It has been reported that patients within 1 month post-transplant have lower MPA AUC_0-12_ than patients between 3 and 6 months post-transplant [7, 11]. Moreover, van Hest et al. [15] reported that POD was a significant factor affecting the pharmacokinetics of MPA. In our present study, the results of multiple comparisons of MPA oral clearance between four groups classified by POD proved that MPA oral clearance on POD < 31 was higher than that of POD ≥ 31 (Fig. 1). Furthermore, estimated AUC_0-12_ on POD < 31 did not exhibit better precision and reliability than that on POD ≥ 31 when Method A was applied (RMSE, R^2^, and q^2^ for POD < 31 or POD ≥ 31 were 6.7, 0.78, and 0.73 or 4.2, 0.90, and 0.89, respectively (data not shown)). We also compared MPA clearance between POD < 91 and POD ≥ 91. MPA clearance was lower in POD < 91 compared with that in POD ≥ 91 (17.2 [8.6–32.7] v.s. 12.5 [6.4–34.1], *P* = 0.0009). However, there was no significant difference in MPA clearance between POD31-90 and POD ≥ 91 (POD31-90: 14.3 [8.6–19.8] v.s. POD ≥ 91: 12.5 [6.4–34.1], *p* = 0.16). Moreover, LSSs classified by PODs into POD < 91 and POD ≥ 91 was not better than Method B (POD < 91: R^2^ = 0.86, RMSE = 5.86, q^2^ = 0.82; POD ≥ 91: R^2^ = 0.91, RMSE = 3.54, q^2^ = 0.88). Therefore, data from both these previous studies and our study support our opinion that estimation equations for MPA AUC_0-12_ with built-in consideration of POD should have better precision and reliability. This is because there is a difference in MPA pharmacokinetics between POD < 31 and POD ≥ 31.

This study has some limitations. First, measured serum MPA concentration includes serum acyl-glucuronide metabolite (AcMPAG) concentration because serum MPA concentration was measured by PETINIA, and the antibody used in PETINIA cross-reacts with AcMPAG [16]. Therefore, the universality of LLS developed in the present study may be limited. Second, the contribution of concomitant drugs is not completely overseen by the present study. However, in LSS by Method B, we performed multiple comparison testing of delta AUC between groups classified by concomitant drug usage, proving there is no difference in delta AUC between groups (Additional files 1, 2, and 3). Therefore, LSSs devised in this study seem to be slightly influenced by the concomitant use of drugs such as quinolone, proton pump inhibitors, and valganciclovir. Third, we cannot explain the mechanism by which mycophenolate clearance changed according to POD. Because of small number of patients involved in the present study, some MPA concentration profiles has been obtained from same patients.

Pawinski et al. [17] reported that AUC_0-12_ estimation comprising three blood-sampling points (0, 0.5, and 2.0 h) may provide good prediction of MPA AUC_0-12_ in renal transplant patients receiving concomitant tacrolimus. However, we could not find literature on LSS that satisfied the following conditions: i) concomitant use of tacrolimus as a calcineurin inhibitor, ii) containing data within 1 month after renal transplantation, and iii) determination of MPA using the PETINIA method. We evaluated the LSS developed by Pawinski et al., which had been well analyzed and meets two conditions (concomitant with tacrolimus and containing data within 1 month after renal transplantation). However, using this equation, we could not obtain a good correlation between the estimated and measured MPA AUCs in our study population (y = 0.74x + 8.32, R^2^ = 0.47, Additional file 4). The reason for this might be explained as follows: i) patients in our study were concomitantly administered once daily prolonged release tacrolimus; ii) our study population included many early post-transplant patients; and iii) the frequency of *UGT1A9* (a metabolic enzyme of MPA) variants (UGT1A9*1, UGT1A9*1c, and UGT1A9*3) varies in the Caucasian, African, and Asian populations [18].

It has been reported that the MPA AUC_0-12_, measured by PETINIA method, are overestimated in comparison with MPA concentrations measured by high performance liquid chromatograph (HPLC) method [19]. Miura et al. compared LSS on the POD28 and 1 year after transplantation in Japanese kidney transplant patients. In that study, the values of MPA AUC_0-12_ (mean [standard deviation (SD)]) measured by HPLC method, were 63.9 [28.9] on the POD 28 and 58.1 [24.3] on 1 year after transplantation, respectively [20, 21]. On the other hand, in the present study, the values of MPA AUC_0-12_ (mean [SD]) were 44.6 [14.4] on the POD 21-28 (*n* = 10) and 45.2 [10.2] on about 1 year after transplantation (POD 345-401), respectively (data not shown). Although dose of MMF in our study was similar to that in their study, the mean of MPA AUC_0-12_ in our study population were lower than that by Miura et al.. In our study, patients was administered prolonged-release tacrolimus concomitantly, whereas in the study of Miura et al., tacrolimus administered to patients was not prolonged-release formulation. Although controversy remains about the interaction between MMF and tacrolimus, prolonged-release tacrolimus formulation might less effect on pharmacokinetics of MPA, since the formulation decreases C_max_ of tacrolimus. Therefore, the difference in formulation of tacrolimus might be the reason for the difference of the MPA AUC_0-12_ between study of Miura et al. and our present study.

Yamaguchi et al. [22] reported the estimation equations for MPA AUC_0-12_ in the Japanese population. However, the usefulness of the estimation equation developed by Yamaguchi et al. was limited because the correlation between the measured and estimated AUC_0-12_ at 1 and 3 months after renal transplantation was not good enough and cross validation of the estimation equation was not performed. In this study, it was demonstrated that our estimation equation was better correlation than that of Yamaguchi et al. and we validated its reliability by cross validation.

In the present study, LSS consisting of four timed samples within 6 h after administration provided accurate and reliable estimation of MPA AUC_0-12_ and was best among the verified estimation equations (Additional file 5). It is known that MPA plasma concentration profile shows a secondary peak at around 6 h after administration because of enterohepatic circulation [16] and its mean contribution to the overall MPA AUC_0-12_ is 37% (10–61%) [23]. Therefore, MPA blood concentration at around 6 h after administration should be important for estimating MPA AUC_0-12_. Measurement of MPA AUC_0-12_ by using 10–12 timed blood samples is a burden on both the patient and medical staff, requiring laboratory resources, considerable quantities of patients’ blood, and a minimum 12 h stay in hospital. Therefore, LSSs that developed by this study, not only Method B but also Method A, will reduce the burden on both patients and medical staff and measurement costs.

## Conclusions

This study suggested that LSSs with consideration of POD provide more accurate and reliable estimations of MPA AUC_0-12_ in Japanese renal transplant patients receiving concomitant tacrolimus therapy.

## Additional files

Additional file 1: Multiple comparison of delta AUC_0-12_ between five groups classified according to concomitant drug usage (all patients). (PPTX 61 kb)

Additional file 2: Multiple comparison of delta AUC_﻿0-12﻿_ between three groups classified according to concomitant drug usage (POD<31). (PPTX 53 kb)

Additional file 3: Multiple comparison of delta AUC_0-12_ between five groups classified according to concomitant drug usage (POD≥31). (PPTX 56 kb)

Additional file 4: Correlation between measured and estimated AUC_0-12_ estimated by using Pawinski’s estimation formula. (PPTX 56 kb)

Additional file 5: Equations obtained using limited sampling time points for the prediction of MPA AUC_0-12_, Correlations with measured MPA AUC_0-12_ and RMSE of estimated AUC_0-12_. (XLSX 11 kb)

## Abbreviations

AcMPAG: Acyl-glucuronide metabolite
AUC: Area under the concentration-time curve
LOOCV: Leave-one-out cross-validation
LSSs: Limited sampling strategies
MMF: Mycophenolate mofetil
MPA: Mycophenolic acid
PETINIA: Particle-enhanced turbidimetric inhibition immunoassay
POD: Postoperative days
RMSE: Root-mean-square error
