# Supplementary material for: Usefulness of limited sampling strategy for mycophenolic acid area under the curve considering postoperative days in living-donor renal transplant recipients with concomitant prolonged-release tacrolimus
Source: J Pharm Health Care Sci. 2017 Jun 24;3:17. doi: 10.1186/s40780-017-0086-7 (PMC5483304; doi:10.1186/s40780-017-0086-7)
Supplement: Supplementary file 1 — Multiple comparison of delta AUC0-12 between five groups classified according to concomitant drug usage (all patients). (PPTX 61 kb) [file 40780_2017_86_MOESM1_ESM.pptx]

## Slide 1
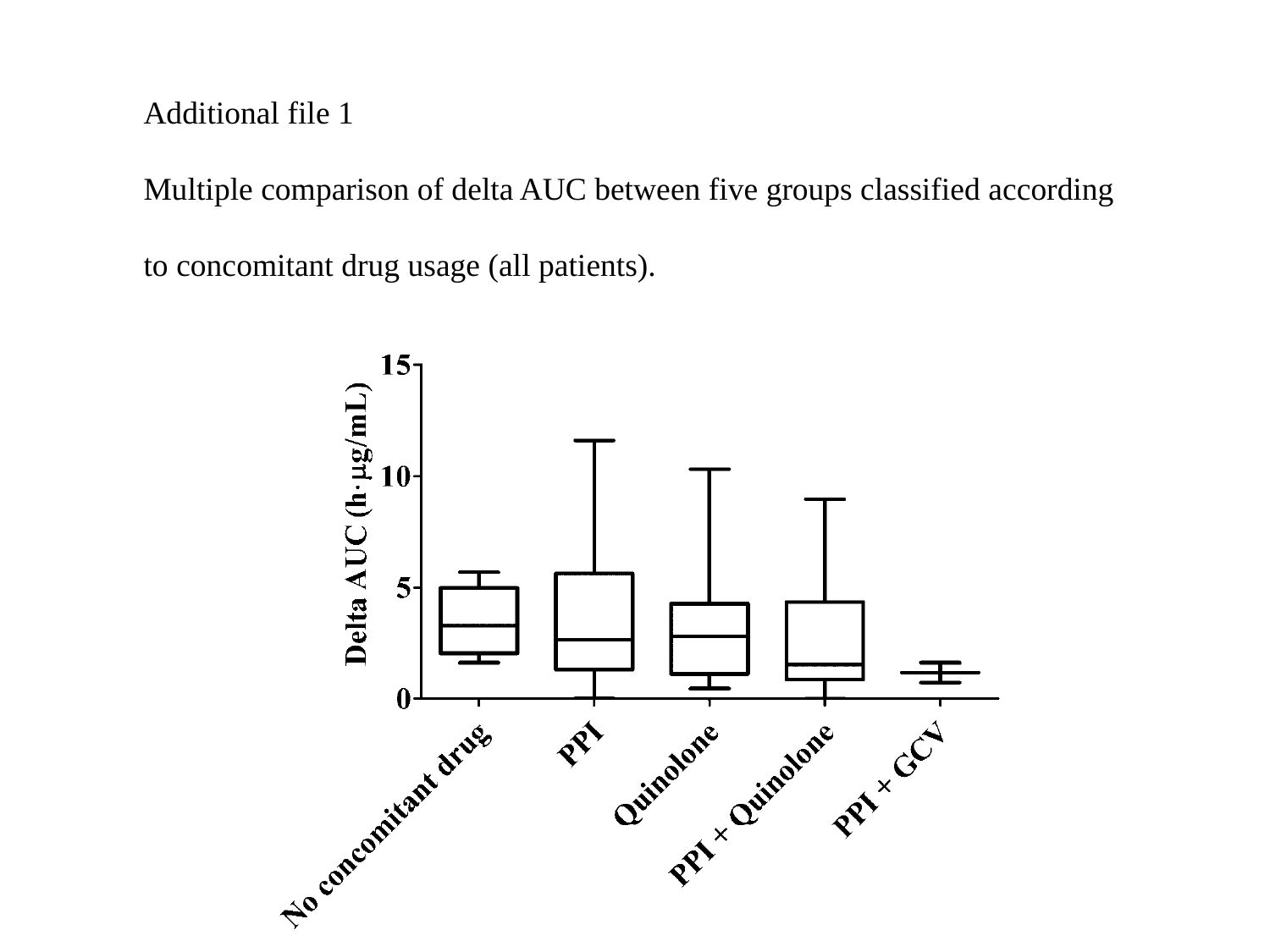

Additional file 1
Multiple comparison of delta AUC between five groups classified according to concomitant drug usage (all patients).
