# Supplementary material for: Usefulness of limited sampling strategy for mycophenolic acid area under the curve considering postoperative days in living-donor renal transplant recipients with concomitant prolonged-release tacrolimus
Source: J Pharm Health Care Sci. 2017 Jun 24;3:17. doi: 10.1186/s40780-017-0086-7 (PMC5483304; doi:10.1186/s40780-017-0086-7)
Supplement: Supplementary file 3 — Multiple comparison of delta AUC0-12 between five groups classified according to concomitant drug usage (POD≥31). (PPTX 56 kb) [file 40780_2017_86_MOESM3_ESM.pptx]

## Slide 1
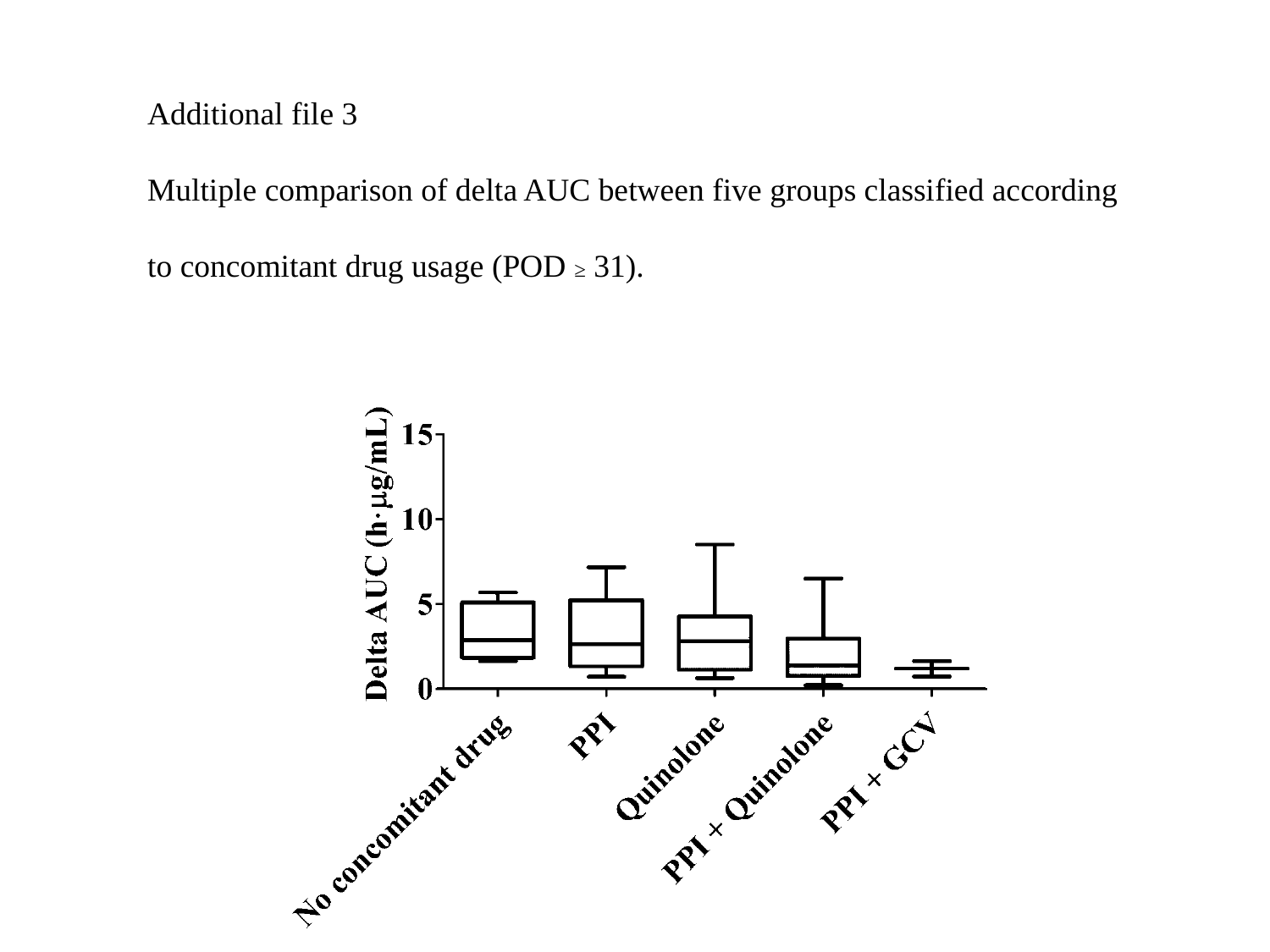

Additional file 3
Multiple comparison of delta AUC between five groups classified according to concomitant drug usage (POD ≥ 31).
