# Supplementary material for: Usefulness of limited sampling strategy for mycophenolic acid area under the curve considering postoperative days in living-donor renal transplant recipients with concomitant prolonged-release tacrolimus
Source: J Pharm Health Care Sci. 2017 Jun 24;3:17. doi: 10.1186/s40780-017-0086-7 (PMC5483304; doi:10.1186/s40780-017-0086-7)
Supplement: Supplementary file 4 — Correlation between measured and estimated AUC0-12 estimated by using Pawinski’s estimation formula. (PPTX 56 kb) [file 40780_2017_86_MOESM4_ESM.pptx]

## Slide 1
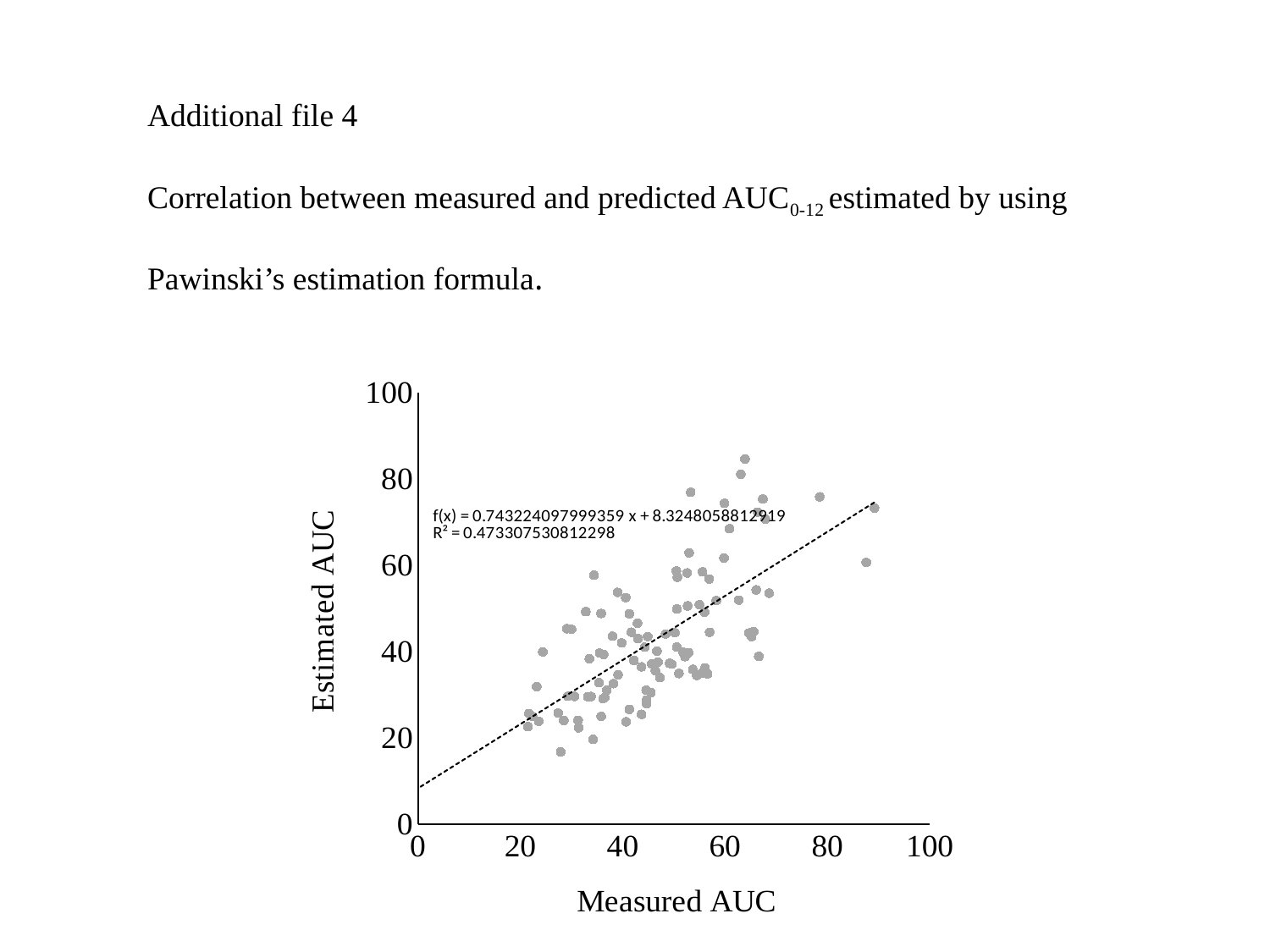

Additional file 4
Correlation between measured and predicted AUC0-12 estimated by using Pawinski’s estimation formula.
### Chart
| Category | AUC (Pawinskiらの推定式) |
|---|---|
